# Representing Diversity in the Dish: Using Patient-Derived *in Vitro* Models to Recreate the Heterogeneity of Neurological Disease

**DOI:** 10.3389/fnins.2018.00056

**Published:** 2018-02-09

**Authors:** Layla T. Ghaffari, Alexander Starr, Andrew T. Nelson, Rita Sattler

**Affiliations:** Department of Neurobiology, Barrow Neurological Institute, Dignity Health—St. Joseph's Hospital and Medical Center, Phoenix, AZ, United States

**Keywords:** IPSC, iNeuron, ALS, FTD, Alzheimer's disease, Parkinson's disease, Huntington's disease

## Abstract

Neurological diseases, including dementias such as Alzheimer's disease (AD) and fronto-temporal dementia (FTD) and degenerative motor neuron diseases such as amyotrophic lateral sclerosis (ALS), are responsible for an increasing fraction of worldwide fatalities. Researching these heterogeneous diseases requires models that endogenously express the full array of genetic and epigenetic factors which may influence disease development in both familial and sporadic patients. Here, we discuss the two primary methods of developing patient-derived neurons and glia to model neurodegenerative disease: reprogramming somatic cells into induced pluripotent stem cells (iPSCs), which are differentiated into neurons or glial cells, or directly converting (DC) somatic cells into neurons (iNeurons) or glial cells. Distinct differentiation techniques for both models result in a variety of neuronal and glial cell types, which have been successful in displaying unique hallmarks of a variety of neurological diseases. Yield, length of differentiation, ease of genetic manipulation, expression of cell-specific markers, and recapitulation of disease pathogenesis are presented as determining factors in how these methods may be used separately or together to ascertain mechanisms of disease and identify therapeutics for distinct patient populations or for specific individuals in personalized medicine projects.

## Introduction

Despite recent, rapid advancement in our understanding of biology and genetics, neurological disease remains a persistent and fatal threat to human health. The World Health Organization (WHO) estimates that the fraction of worldwide deaths attributable to neurological conditions increased 89% from 2000 to 2015 (WHO, 2017). Alzheimer's disease (AD) and other dementias make up the vast majority of these fatalities, but Parkinson's disease (PD), epilepsy, Huntington's disease (HD), and motor neuron diseases such as amyotrophic lateral sclerosis (ALS) are each responsible for tens of thousands of deaths per year. The variety of symptoms and heterogeneity of affected individuals make diseases of the central nervous system (CNS) difficult to diagnose, study, and treat (Kim and Jeon, 2016; Van Cauwenberghe et al., 2016; Van Damme et al., 2017).

Staining of post-mortem tissue remains the only means of officially diagnosing several neurological diseases, and studying the mechanisms behind defining proteinopathies is difficult in this static state. The postmitotic nature of neurons and difficulty of tissue access make biopsies of the CNS infeasible, and the blood-brain barrier isolates many biochemical signals of disease progression from peripheral biofluids. Thus, in order to properly study pre-symptomatic disease or disease progression mechanisms in patients, we must rely on human *in vitro* models.

For genetically inherited disease, where a specific causal mutation has been identified, cellular models of disease can be created by genetic manipulations (overexpression, siRNA knockout, CRISPR/Cas9 knock-in, or knock-out) of cells lines or primary rodent cell cultures. Alternatively, primary rodent cell cultures can be generated from existing animal models of disease (e.g., mutant SOD1 mouse model of ALS, R2/6 mouse model of Huntington's disease). These approaches do come with limitations. For example, overexpression models can simultaneously mask subtle disease phenotypes while falsely exaggerating or creating entirely non-biological pathology. While these models may be used for early studies into disease mechanisms, true biological representation, especially for potential therapeutic development, requires endogenous expression of known genetic mutations and their genetic interactors, which emphasizes the need for patient-derived models.

Patient-derived models become even more necessary for studying sporadic disease, as there is no way of definitively recapitulating the disease in a non-diseased organism. The genetic and epigenetic heterogeneity of sporadic patients may make specific causative mutations statistically undetectable, or a confluence of small changes may be required to cause disease phenotypes. Such complexity would be impossible to recapitulate through genetic manipulation of a non-diseased cell, even if the full spectrum of contributory expression was identified. Since sporadic patients make up the majority of patients in diseases such as ALS, Frontotemporal Dementia (FTD), and AD (Kim and Jeon, 2016; Van Cauwenberghe et al., 2016; Van Damme et al., 2017), sporadic patient-derived cells offer the most widely applicable tool for studies of disease mechanisms and therapeutic screens.

**Figure 1:**
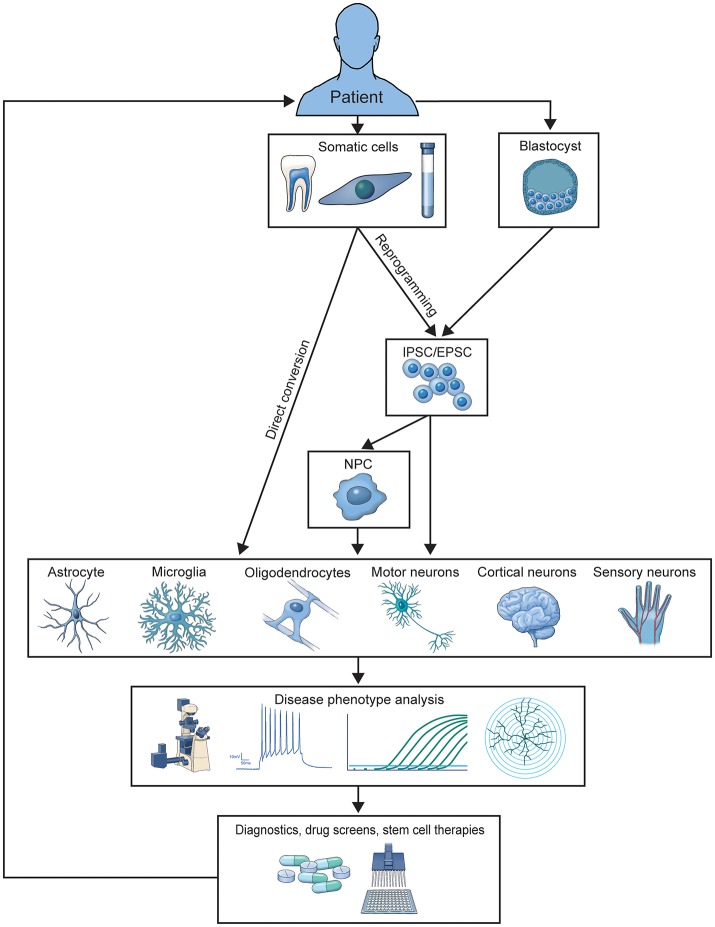
Illustration of the generation and application of patient-derived models. Patient cells are used to generate iPSC or DC-based *in vitro* models of familial or sporadic disease. These models are studied to elucidate disease-contributing mechanisms and screen preclinical therapeutics, which can be translated into treatments for donor patient populations.

Two primary methods for generating patient-derived cells have emerged. Somatic cells can be reprogrammed into induced pluripotent stem cells (iPSCs) and then differentiated into the desired cell type or directly converted (DC) into the desired cell type (Figure 1). Each method has been expanded over the last decade for differentiation into a wide variety of CNS cells used to study and treat neurological disease. Here, we review how these models have developed over the last 10 years, the methods used to differentiate them, the specific cell types those methods produce, and how disease has been studied in these models. We also highlight how human culture models are providing us with new mechanistic insights that were previously unattainable with postmortem patient tissue analysis or animal models of disease, and discuss the current limitations of *in vitro* modeling of CNS disease.

## iPSC overview

Early experiments in using stem cells to model neurons were largely developmental and relied upon the differentiation of embryonic stem cells (ESCs) (Thomson et al., 1998). Because these cells were not patient-derived, they were either cultured from mutant mouse embryos (Di Giorgio et al., 2007) or were healthy ESCs transfected to overexpress known mutants (Karumbayaram et al., 2009). These models encounter the same setbacks as all over-expressions, and thus offer little improvement over simpler models. Recently, preimplantation genetic diagnosis (PGD) has offered an opportunity for ESCs to be generated from embryos known to carry disease causing mutations, namely the hexanucleotide expansion of C9orf72 (C9) (Cohen-Hadad et al., 2016). Methylation analysis of motor neurons differentiated from these ESCs compared to adult C9 patient-derived iPSCs revealed hypermethylation of the iPSCs, which would suggest that ESCs provide a better representation of patient epigenetics. However, ethical concerns, the limited availability of endogenously mutated ESCs, and their inability to be used to model sporadic disease calls for the ability to generate human-derived *in vitro* models from adult patients. Experiments which showed that ESC fusion could confer pluripotency to fibroblasts suggested that certain cellular products might induce pluripotency even in an adult somatic cell (Cowan et al., 2005).

Since the initial, groundbreaking discovery of four transcription factors (Oct3/4, Sox2, c-Myc, and Klf4) capable of converting adult mouse (Takahashi and Yamanaka, 2006) and human (Yu et al., 2007) fibroblasts into pluripotent stem cells, a number of new methods for reprogramming somatic cells have been developed, including the use of various viral and chemical transfection methods to express optimized pluripotency factors. iPSCs can be generated from a wide variety of somatic cells, including skin, peripheral blood mononuclear cells, and hair, which enables non- or minimally-invasive collection of patient samples to generate models of their disease (Egusa et al., 2010).

## iPSC differentiation methods

Prior to the choice of which specific neural cell to generate, the basic method of differentiation must be chosen. Many protocols rely on the addition of small molecules and growth factors to first induce embryoid bodies followed by neural rosettes (Elkabetz et al., 2008), or procede directly from iPSCs to neuroprogenitor cells (NPCs) via dual-SMAD signaling inhibition (Chambers et al., 2009). NPCs can be maintained and further differentiated and matured using growth factors. NPCs can be infinitely expanded, frozen down, and thawed to provide a mid-differentiation starting point, which reduces derivation time by ~30% (Brafman, 2015).

Some differentiation protocols combine small molecules and growth factors with transfection of lineage-specific transcription factors, in a manner similar to direct conversion. For example, Wen et al. used transcription factors established by Son et al. for direct conversion to generate iPSC induced motor neurons for the study of repeat associated non-ATG (RAN) translated dipeptide repeat proteins (DPRs) in C9 ALS (Son et al., 2011; Wen et al., 2014). Other groups have reported that transcription factors may be used to more quickly generate very pure cultures of dopaminergic and GABAergic neurons (Theka et al., 2013; Yang et al., 2017). Neural progenitor cells (NPCs) have been derived using only small molecules (Reinhardt et al., 2013), and Li et al. discovered that microRNA 199a could induce angiogenesis in iPSCs, which may indicate that small-molecule only and microRNA based differentiation protocols, which have been used more widely in direct conversion, could be developed for iPSCs (Li Z. et al., 2015).

Another differentiation alternative attempts to recreate the physics of the biological environment by differentiating iPSCs in structured organoids. Unique basal hydrogels, custom culture chambers, and physical agitation can be used to encourage iPSCs to differentiate into a specific three-dimensional structure (Lindborg et al., 2016). This may lead to greater representation of brain-layer formation, which may improve cell maturity, stimulate expression of disease phenotypes, and provide a more accurate model for drug screens (Raja et al., 2016). In addition, organoids can provide a method of modeling diseases which are difficult to recreate in animal models, such as microencephaly (Lancaster et al., 2013).

## iPSC differentiated cell types

Human iPSCs have been successfully differentiated into a number of disease-relevant cell types, as verified by morphology, gene expression profiles, and cell-specific protein expression. Many differentiation protocols begin with the development of (NPCs). These have been used primarily for the development of therapeutic neuron replacement (Nicaise et al., 2017). Similarly, oligodendrocyte precursors have been used to drive implanted cells to an oligodendrocyte fate for remyelination therapies (Douvaras et al., 2014; Kawabata et al., 2016). Differentiation beyond precursors yields neurons and glia with varying efficiencies and purity. Variations in small molecule and growth factor selection, maturation duration, and culture conditions continue to be optimized.

### iPSC brain-native neuronal subtype differentiation

iPSCs have been differentiated into a number of brain-native neuronal subtypes. Cortical glutamatergic neurons can be generated by initializing neuralization using retinoids and SMAD inhibition. This generates cerebral cortex stem cells, which mature over 60 days into neurons representing the cortical layers (Shi et al., 2012). Viral expression of LMX1A in NPC cultured on PA6 stromal cell yields dopaminergic (DA) neurons (Sanchez-Danes et al., 2012). The combined effect of virally expressed ASCL1, NURR1, and LMX1A can generate iPSC DA neurons without the need for PA6 co-culture (Theka et al., 2013). Inhibitory GABA-ergic neurons can also be derived via viral induction of ASCL and DLX2 (Yang et al., 2017). These are but a few examples of the wide variety of neuronal subtypes created from iPSCs from an even wider array of protocols.

### iPSC spinal motor neuron differentiation

Neurons native to the spinal cord and periphery, including motor and sensory neurons, can be generated readily from iPSCs. iPSC motor neurons (iPSCMNs) are generated by treatment with retinoic acid and sonic hedgehog (SHH), which is required for floor-plate differentiation (Ericson et al., 1996). iPSCMNs express HB9, Islet-1, and ChAT, which indicate a mature cholinergic maturation (Dimos et al., 2008). Peripheral nerves have also been successfully modeled using iPSCs. Nociceptor precursor cells can be generated from stem cells in 24 days, transfected to overexpress Neurog1, and matured into capsaicin-sensitive nociceptors to study nerve pain disorders (Boisvert et al., 2015).

### iPSC glial differentiation

Glia can also be recreated from iPSCs, including astrocytes, oligodendrocytes, and microglia. These can be studied as mono-cultures or co-cultured with neurons to study non-cell autonomous effects on disease, be they contributory or compensatory.

Astrocytes are necessary to provide neurotrophic support and maintain healthy synaptic conditions. Krencik et al first reported the differentiation of iPSCs into astrocytes (Krencik et al., 2011). The resulting cells were GFAP positive, S100B positive, and took 180 days to differentiate, because astrocyte differentiation occurs after neuronal differentiation (Emdad et al., 2012). Differentiating iPSCs into neural stem cells before astrocyte differentiation reduced the generation time to 6 weeks (Shaltouki et al., 2013). Fluorescence-activated cell sorting (FACS) of differentiated iPSCs virally transfected to express GFP under a GFAP promoter allows for the selection of a purely astrocytic culture and reduces “noise” from neurons and other glia remaining from early periods of differentiation (Zhang et al., 2016).

In addition to the use of oligodendrocyte precursors (OPCs) in therapeutic transplantation, mature iPSC-derived oligodendrocytes have been generated. Early reports suggested that iPSCs might contain some inherent factor which prevented oligodendrocyte maturation (Tokumoto et al., 2010), however, a low yield, high purity, three-month protocol was soon developed (Hu et al., 2009). These mature oligodendrocytes were capable of generating myelin sheaths. Oligodendrocyte precursors can be matured by co-culture with dorsal root ganglion (DRG) neurons and will myelinate them *in vitro* (Czepiel et al., 2011). Douvaras et al. used a protocol similar to iPSMN differentiation, including adherent dual SMAD inhibition and early caudalization and ventralization, to increase OPC yield and shorten differentiation time to 75 days (Douvaras et al., 2014).

Microglia derive from macrophages but do not gain certain distinct features until they develop in concert with the nervous system. Early attempts to generate microglia from iPSCs resulted in cells called microglia-like macrophages, and while they did not express typical microglial morphology, they were positive for IBA1, CD45 and CD11b (Muffat et al., 2016). When microglia-like macrophages were co-cultured with iPSCNs, they exhibited a more mature microglial morphology and phagocytic behavior (Haenseler et al., 2017a). Recently, Douvaras et al. reported a method to develop mature, ramifying microglia without co-culturing (Douvaras et al., 2017).

### iPSC differentiation into neural-interfacing cell types

Non-neuronal cells relevant to neurological disease have also been derived. iPSCs can be differentiated into myoblasts, which form contracting, electrically and chemically responsive myotubes that are capable of forming neuromuscular junctions (NMJs) (Demestre et al., 2015). Additionally, endothelial cells can be generated to model the blood-brain-barrier, which is suggested to be compromised in neurodegeneration (Lippmann et al., 2012).

With the ability to differentiate into the full complement of neurons, glia, and neural-interfacing cells, iPSCs provide a powerful model for the study of disease in individual cells, in co-culture, and in three-dimensional reconstructions of complex tissues. Many examples of subtype-specific differentiation methods focus on the expression of specific marker proteins. While this is an important indicator of an individual cell type, more parameters can and should be considered when evaluating differentiation protocols. For example, positive expression of marker proteins should be correlated with the lack of expression of specific marker proteins of similar, yet distinct, cell types, e.g., cortical forebrain neurons should lack markers of motor neurons. In addition, gene expression profiles should be compared to data from either primary murine cultures of the same cell type or isolated cell types from postmortem brain tissues. Similarly, morphology and electrophysiological activity should be included in the cell characterization. This will increase confidence in claims of cell-type specificity and also provide more experimental readouts to use as standards for maintaining consistent models across multiple rounds of differentiation.

Additional steps may be employed to reduce differentiation variability and to ensure disease relevance of observed deficits. While the use of multiple disease and control cell lines controls for the variability between individual human subjects, multiple differentiations of the same cell line accounts for variability between differentiations. In addition, CRISPR-Cas9 mediated gene editing allows for the correction of disease-causing mutations (Cong et al., 2013). This provides the possibility of creating isogenic control iPSC lines, which genetically and epigenetically match patient derived lines, except for the corrected mutation. If this correction alleviates deficits, it supports the association between the gene mutation-dependent phenotypes and actual disease pathology. This can be strengthened further by using the same technique to introduce known mutations in healthy control iPSCs, creating a positive control. Unfortunately, isogenic controls cannot be created to study sporadic disease which, as mentioned above, represents the majority population of most neurodegenerative diseases.

## iPSC models of disease

### Amyotrophic lateral sclerosis/frontotemporal dementia

**Figure 2:**
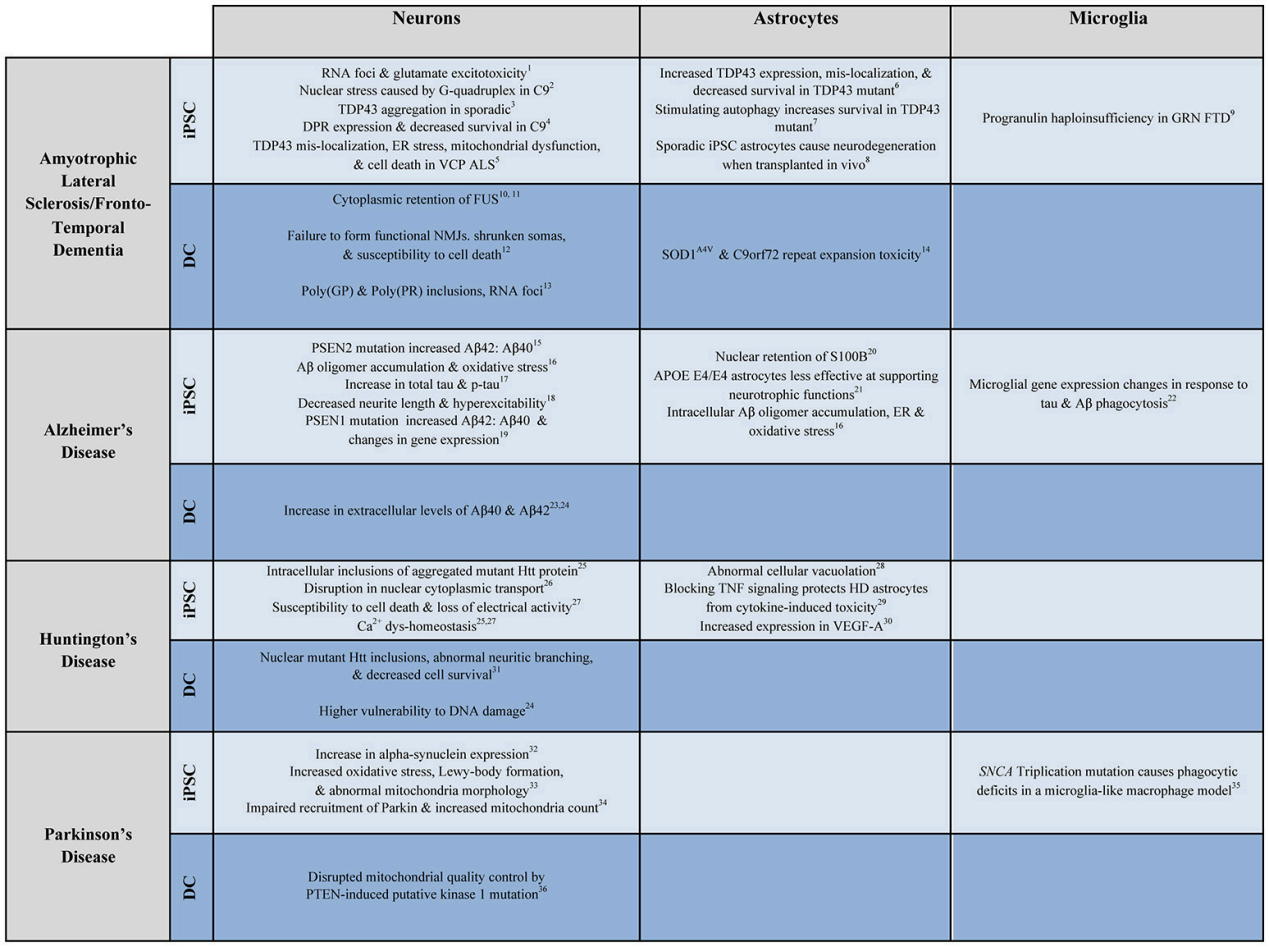
Examples of patient-derived *in vitro* models of neurological disease. Select examples of how patient-derived iPSC and DC neurons, astrocytes, and microglia have been used to model ALS/FTD, AD, HD, and PD. Numbers correspond to the following references: 1. Donnelly et al., 2013; 2. Wang et al., 2014; 3. Burkhardt et al., 2013; 4. Wen et al., 2014; 5. Hall et al., 2017; 6. Serio et al., 2013; 7. Barmada et al., 2014; 8. Qian et al., 2017; 9. Almeida et al., 2012; 10. Liu et al., 2016; 11. Lim et al., 2016; 12. Son et al., 2011; 13. Su et al., 2014; 14. Meyer et al., 2014; 15. Yagi et al., 2011; 16. Kondo et al., 2013; 17. Muratore et al., 2014; 18. Balez et al., 2016; 19. Sproul et al., 2014; 20. Jones et al., 2017; 21. Zhao et al., 2017; 22. Abud et al., 2017; 23. Hu et al., 2015; 24. Hou et al., 2017; 25. Nekrasov et al., 2016; 26. Grima et al., 2017; 27. Mattis et al., 2015; 28. Juopperi et al., 2012; 29. Hsiao et al., 2014; 30. Hsiao et al., 2015; 31. Liu et al., 2013; 32. Devine et al., 2011; 33. Imaizumi et al., 2012; 34. Seibler et al., 2011; 35. Haenseler et al., 2017b; 36. Puschmann et al., 2017.

iPSCs have been used extensively to model neurological disease (Figure 2). ALS, a fatal motor neuron disease, and FTD, a neurodegenerative dementia, appear to exist on a genetic spectrum of disease, with several shared genetic causes and identifying pathologies. It is in this spectrum that the widest variety of iPSC modeling has been employed. iPSCMNs are the most widely used, and have been shown to demonstrate the specific pathology and physiology of ALS subtypes. Dimos et al. were the first to report the generation of iPSCMNs from an ALS patient that expressed the motor neuron-specific transcription factors HB9 and Islet 1 (Dimos et al., 2008). It was not until four years later that actual disease phenotypes were first shown in TDP-43 ALS patient-derivedmotor neurons. Egawa and colleagues differentiated iPSCMNs from three ALS patients carrying three distinct TDP-43 mutations and showed that, among other deficits, the diseased motor neurons exhibited hallmark TDP-43 aggregates similar to those found in postmortem ALS brain tissues (Egawa et al., 2012). The authors were able to rescue these phenotypes with a histone acetyltransferase inhibitor, providing first evidence that patient-derived iPSCMN culture models could be used for drug screening purposes. iPSC modeling of FTD soon followed when Almeida and colleagues generated iPSC neurons and microglial cells from FTD patients carrying a mutation in progranulin (PGRN) (Almeida et al., 2012). The differentiated human cells confirmed the presence of PGRN haploinsufficiency, in addition to aiding in the new discovery of mutant PGRN-specific deficits of certain protein kinase pathways. When the C9orf72 hexanucleotide expansion was discovered as the most common shared mutation in ALS/FTD disease spectrum in 2011(DeJesus-Hernandez et al., 2011; Renton et al., 2011), the fastest way to study disease mechanisms was via iPSCs, as the generation of the repeat expansion in mouse models proved to be difficult. Several laboratories shortly thereafter provided the first evidence of the three major disease mechanisms of mutant C9orf72 using iPSC neurons: haploinsuffiency, RNA foci formation, and repeat-associated non-ATG (RAN) translation (Almeida et al., 2013; Donnelly et al., 2013; Lagier-Tourenne et al., 2013; Sareen et al., 2013). These studies also revealed disease-mediated aberrant gene expression and neuronal function in patient iPSCs. Numerous follow up studies examining both neuronal and glial iPSC culture models have since been used to further investigate how mutant C9orf72 leads to neuronal degeneration, ranging from studies on nucleocytoplasmic trafficking deficits (Freibaum et al., 2015; Zhang et al., 2015; Lopez-Gonzalez et al., 2016; Westergard et al., 2016) to the toxic potential of RAN dipeptides, as recently reviewed (Selvaraj et al., 2017).

Other familial ALS mutation phenotypes, such as valosin-containing protein (VCP) (Hall et al., 2017), TAR DNA-binding protein 43 (TDP-43) (Serio et al., 2013; Barmada et al., 2014), and superoxide dismutase (SOD1) (Bhinge et al., 2017), have been recapitulated in iPSCs showing characteristic proteinopathies, and endoplasmic reticulum (ER) and oxidative stress. To determine which disease pathologies in the model are caused primarily by the mutation, isogenic corrections of known genetic causes, such as SOD1, have been generated, and were shown to successfully rescue the mutant phenotypes, such as previously described hyperexcitability in SOD1 mutant iPSCMNs (Wainger et al., 2014; Bhinge et al., 2017).

Importantly, considering sporadic disease represents 90% of ALS patients, sporadic iPSCs have also been differentiated into neurons and were shown to exhibit TDP-43 pathology (Burkhardt et al., 2013; Qian et al., 2017). Furthermore, gene expression profiling of sporadic ALS iPSCMNs suggested deficits in mitochondrial function (Alves et al., 2015). More studies are required to better understand the pathogenesis of sporadic disease, and human cell culture models are currently the only way to gain insight into what triggers and propagates sporadic ALS and FTD.

Limited studies have addressed the role of glial cells in ALS/FTD, although both ALS and FTD patient-derived iPSC astrocytes have been employed to study the non-cell autonomous mechanisms of neurodegeneration. Interestingly, while studies using SOD1 mutant mouse models and primary cultures generated from these mice unequivocally demonstrated that mutant astrocytes trigger motor neuron degeneration, iPSC astrocytes differentiated from ALS patients carrying a TDP-43 mutation, while exhibiting TDP-43 mislocalization themselves, did not affect motor neuron survival in a co-culture model (Serio et al., 2013). On the other hand, iPSC astrocyte-conditioned media derived from C9orf72 patients leads to impairments of iPSC motor neuron autophagy (Madill et al., 2017). Similarly, iPSC astrocytes from mutant VCP patients affect motor neuron survival *in vitro* (Hall et al., 2017). In addition, transplanting sporadic ALS iPSCs into the spinal cord of WT mice resulted in differentiation into astrocytes and triggered a loss of neurons in the spinal cord, with accompanying movement deficits (Qian et al., 2017).

Even fewer studies to date have generated and characterized iPSC-derived oligodendrocytes. Ferraiuolo and colleagues used both sporadic and fALS patient-derived oligodendrocytes to show that, similar to astrocytes, diseased oligodendrocytes affect motor neuron survival (Ferraiuolo et al., 2016). At the same time, Livesey and colleagues studied the maturation and electrophysiological properties of C9orf72 patient-derived iPSC oligodendrocytes and did not observe any obvious impairments or alterations of these cells when grown in mono-cultures (Livesey et al., 2016).

The only study describing iPSC microglial cells from ALS or FTD patients was reported by Almeida et al. who showed that PGRN FTD patient microglial cells exhibit progranulin haploinsufficiency, but do not show the deficits in serine/threonine kinase S6K2 observed in iPSC neurons from the same patients (Almeida et al., 2012).

### Alzheimer's disease

The most common dementia, Alzheimer's disease (AD), has been examined extensively in patient-derived iPSCs, and these studies been thoroughly reviewed (Devineni et al., 2016; Arber et al., 2017; Robbins and Price, 2017; Tong et al., 2017). One of the first reports using patient-derived iPSC neurons to study mechanisms of AD examined the pathogenesis of familial Presenilin 1 (PS1) and Presenilin 2 (PS2) (Yagi et al., 2011). The mutant iPSC neurons exhibited increased amyloid β 42 secretion, which was inhibited by a γ-secretase inhibitor. This study was followed by a report from Israel and colleagues who found an increase in Aβ and phosphorylated tau (p-tau) in familial amyloid-β precursor protein (APP) duplication (APP(Dp)) and sporadic iPSC cortical neurons (Israel et al., 2012). Treating patient neurons with β-secretase inhibitor, but not γ-secretase inhibitors, significantly reduced the documented disease phenotypes in the cells, providing new knowledge on the mechanisms of APP pathogenesis.

These early studies were followed by reports using both familial and sporadic AD patient iPSCs differentiated into cortical neurons or, to a limited degree, glial cells to better understand mechanisms of neurodegeneration in AD and for novel therapeutic target investigation. For example (Duan et al., 2014), used sporadic AD patient iPSCs with ApoE3/E4 genotypes and reported not only an increased ratio of Aβ42/40 and responsiveness to γ-secretase inhibitors, but also increased susceptibility to glutamate toxicity with a concomitant increase in free intracellular calcium levels, suggesting that actual neuroprotection could be examined using these patient-derived neurons. Similarly, iPSCs from AD patients carrying an APP mutation (V717I) showed increased expression levels of APP and Aβ, in addition to aberrant β and γ secretase cleavage of APP and increased levels of total and phosphorylated tau (Muratore et al., 2014). Interestingly, the authors were able to show that treatment with specific Aβ antibodies early during the differentiation process rescued the mutant APP phenotypes. This new paradigm of testing drugs in patient-derived cells was strengthened by a study in which apigenin has been tested as a potential anti-inflammatory therapeutic in AD patient-derived neurons co-cultured with activated murine microglial cells. The treatment was successful in reversing morphological deficiencies, reducing hyper-excitability, and protecting against apoptosis (Balez et al., 2016).

Sproul and colleagues studied iPSC derived neural precursor cells (NPC) to understand whether, even at a pre-neuronal stage of development, human cells show AD phenotypes (Sproul et al., 2014). Differentiating iPSCs from mutant PS1 patients into NPCs, the authors observed an increased Aβ42/40 ratio and an aberrant gene expression profile after transcriptome analysis. A select number of gene aberrations were confirmed to be similarly altered in postmortem AD brain tissues. Similar findings were confirmed in PS1 patient iPSC neurons (Mahairaki et al., 2014). Interestingly, generating isogenic iPSC lines using TALENs (TAL Effector Nucleases) technology to insert PS1Δ E9 mutations in otherwise healthy control patient lines lead to the discovery that this mutation represents a toxic gain of function mutation that impairs PS1-dependent γ secretase activity, but not unrelated γ secretase functions (Woodruff et al., 2013).

Few studies thus far have used patient-derived iPSC astrocytes to model Alzheimer's disease. Kondo et al showed that iPSC astrocytes from sporadic AD patients and patients with an (APP)-E693delta mutation displayed ER and oxidative stress, which was alleviated with treatment of docosahexaenoic acid (DHA) (Kondo et al., 2013). A more recent study showed that iPSC astrocytes from familial and sporadic AD patients display significant morphological phenotypes with an overall atrophic profile and mislocalization of astroglial marker proteins, including S100B (Jones et al., 2017). IPSCs astrocytes from normal individuals with APOE ε4/ε4 genotypes were less effective in promoting neuronal survival and synaptogenesis when compared to iPSC astrocytes from normal individuals with APOE ε3/ε3 genotypes (Zhao et al., 2017), illustrating that patient-derived culture models could aid in exploring the role of individual apoE isoforms in AD disease pathogenesis. Finally, iPSC astrocytes differentiated from patients carrying the PSEN1 delta E9 mutation exhibited typical AD pathology—increased β amyloid, altered cytokine release, defective calcium homeostasis, increased oxidative stress and reduced lactate secretion (Oksanen et al., 2017). None of these phenotypes were present in healthy control or gene-corrected isogenic iPSC astrocytes.

Similar to ALS above, only one study to date has used patient-derived microglial cell to study AD disease mechanisms. Abud and colleagues generated iPSC microglial-like cells and showed altered gene expression in response to Aβ fibrils and aberrant phagocytic activity when exposed to brain-derived tau oligomers (Abud et al., 2017). Further work is required to determine whether the observed AD iPSC-derived glial phenotypes contribute to the neuronal disease pathogenesis in a non-cell autonomous manner.

To overcome the observed inability of 2 dimensional iPSC neuron cell culture models to generate extracellular protein aggregations, the AD field has quickly tried to model AD in 3 dimensional (3D) human cell culture systems (reviewed by and Choi et al., 2016; Lee et al., 2017). One of the first published studies using 3D iPSC differentiated neuron cultures showed that only in the 3D environment did the AD neurons display p21-activated kinase-mediated sensing of Aβ oligomers, in addition to the presence of F-actin associated protein phenotypes (Zhang et al., 2014). Choi and colleagues developed a 3D iPSC neuron culture model from familial AD patients (mutant APP and mutant presenilin), which, for the first time, represented a disease model that displayed hallmarks of AD pathology: extracellular deposition of amyloid-β, including amyloid-β plaques, and aggregates of phosphorylated tau, as well as filamentous tau (Choi et al., 2014; Kim et al., 2015). More recent studies have applied the 3D culture model to generate high throughput models for drug screening against tau aggregation (Medda et al., 2016), or to compare efficacy of drug candidates (β or γ secretase inhibitors) in 2D vs. 3D culture systems (Lee et al., 2016). Finally, the generation of brain organoids, initially developed to model human brain development (Lancaster et al., 2013), has been adopted to study mechanisms of AD as well. Raja and colleagues reported that brain organoids from familial AD patients recapitulate AD disease phenotypes and pathologies including amyloid aggregation, hyperphosphorylated tau, and endosome abnormalities, all of which were reduced by treatment with secretase inhibitors (Raja et al., 2016).

### Huntington's disease

Zhang and colleagues were the first group to characterize HD patient-derived iPSC neuronal cells (Zhang et al., 2010). The authors differentiated iPSCs into striatal neurons expressing cell-specific markers such as DARPP-32. The neurons maintained the CAG repeat expansion and showed increased susceptibility to growth factor removal as shown by enhanced caspase activity. The same research team also generated the first gene-corrected isogenic cell lines, which resulted in the rescue of disease phenotypes, including the susceptibility to cell death and mitochondrial abnormalities (An et al., 2012). A large consortium of Huntington's disease researchers reported increased cell death, sensitivity to stressors, increased glutamate toxicity, and reduced sporadic electrical firing in HD patient-derived iPSC striatal-like neurons as characterized by the expression of Map2a/b and Bcl11B (iPSC Consortium, 2012). Interestingly, the severity of several of these phenotypes increased in proportion to the patient's CAG-expansion length in the *HTT* gene. Similar findings were reported by Mattis and colleagues, who reported increased cell death following BDNF withdrawal, but also showed that increased susceptibility to glutamate toxicity could be blocked by NMDA and AMPA receptor inhibitors (Mattis et al., 2015), suggesting novel therapeutic approaches for HD.

Several other potential Huntington's disease pathways have been identified using patient derived iPSC neurons, such as miR196a dysregulation (Cheng et al., 2013), MAPK and Wnt signaling (Szlachcic et al., 2015), the protective potential of A2A adenosine receptor activation (Chiu et al., 2015), and astrocyte-mediated cytokine-induced neuronal cell death (Hsiao et al., 2014). CRISPR-Cas9 gene-corrected isogenic HD patient lines showed rescue of HD phenotypes, such as deficits in mitochondrial respiration (Xu et al., 2017). Most interestingly, though, these isogenic lines showed that gene expression differences between HD and healthy control iPSCs were not present when HD lines were compared to their isogenic control, suggesting that general differences in genetic background could be falsely identified as transcriptomic abnormalities triggered by the disease-causing mutation. Neskarov et al. reported that HD iPSC neurons show increased numbers of lysosomes/autophagosomes and exhibit increased cell death alongside nuclear indentation (Nekrasov et al., 2016). Further nuclear deficits, including nucleoporin aggregation and impaired nucleo-cytoplasmic transport, have recently been demonstrated in HD patient-derived neurons (Grima et al., 2017); similar to what has been described in ALS (Kim and Taylor, 2017). One of the most recent studies showed that previously reported repression of peroxisome proliferator-activated receptor delta (PPAR-δ)—a ligand-gated transcription factor that promotes mitochondrial biogenesis and oxidative metabolism—in an HD mouse model is also found in HD patient-derived iPSC neurons (Dickey et al., 2016, 2017). Indirect activation of PPARγ via the small molecule compounds bexarotene or KD3010 significantly rescued impaired oxidative metabolism in HD neurons.

There have been few reports of iPSC differentiations into non-neuronal cells for HD. Huntington's iPSC astrocytes were shown to express higher levels of vascular endothelial growth factor-A (VEGF-A) (Hsiao et al., 2015) and formed electron-clear vacuoles, which increased during the differentiation period but appeared independently of cellular stressors (Juopperi et al., 2012). To date, patient derived microglia and oligodendrocytes have not been studied in Huntington's disease, but differentiation of HD iPSCs into brain microvascular endothelial cells revealed abnormalities in angiogenesis and blood brain barrier properties (Lim et al., 2017).

In summary, the use of patient-derived iPSC cultures in HD has provided new insight regarding disease pathogenesis and also provided a novel model to perform pre-clinical drug discovery (Tousley and Kegel-Gleason, 2016). A potential additional application of these patient-derived cells lies in the transplantation of stem cells or their derivatives to replace diseased or lost neurons. While not discussed in this review, numerous efforts have been made toward successful transplantation of cells at the iPSC stage or the neural precursor stage in rodents with the hope to create a therapeutic path toward HD patient transplantations (Golas and Sander, 2016; Connor, 2017; Choi et al., 2018).

### Parkinson's disease

Parkinson's disease (PD) has been extensively modeled using patient-derived iPSCs (Cobb et al., 2017). PD is modeled chiefly by the differentiation of iPSCs into dopaminergic (DA) neurons. Park et al. created the first Parkinson's disease iPSC line, together with other neurodegenerative disease lines (Park et al., 2008). Patient-derived DA neurons exhibit the typical pathology of PD: the presence of Lewy bodies and their main component, α-synuclein. This pathology is present in iPSC DA neurons from patients with mutations in the SNCA gene and in patients with mutations in leucine-rich repeat kinase 2 (LRRK2) (Devine et al., 2011; Nguyen et al., 2011) and, rarely, in patients with mutations in parkin (Imaizumi et al., 2012), but not in patients with a mutation in PTEN-induced putative kinase 1 (PINK1) (Jiang et al., 2012). This reflects the pathology as it has been observed in postmortem patient brain tissue. Other disease phenotypes previously observed in either mouse models or autopsy tissue were confirmed in patient-derived familial and sporadic iPSC DA neurons, including mitochondrial deficits and oxidative stress (Byers et al., 2011; Cooper et al., 2012; Hsieh et al., 2016) and lysosomal and autophagy deficits (Mazzulli et al., 2011, 2016; Sanchez-Danes et al., 2012; Schondorf et al., 2014; Fernandes et al., 2016).

Similar to the aforementioned neurodegenerative diseases, researchers are now using genetically corrected patient-derived iPSCs to study PD pathogenesis in order to reduce the potential for general patient-to-patient genetic variability to obscure disease-specific cellular and genetic alterations, and to pinpoint specific disease mechanisms linked to the genetic mutation examined, and not to potential cellular deficits arising from the actual cell differentiation process (Soldner et al., 2011; Arias-Fuenzalida et al., 2017; Qing et al., 2017).

Parkinson's disease has not been studied extensively in iPSC-derived glia; however, Haenseler et al. have recently shown that phagocytosis is impaired in SNCA triplication microglia (Haenseler et al., 2017b).

### Neuroinflammation and neuropsychiatric diseases

Modeling disease with iPSCs extends beyond neurodegeneration. Although much research in de-myelinating disease, such as multiple sclerosis (MS), focuses on differentiating healthy oligodendrocyte precursors for transplantation replacement therapy (Kawabata et al., 2016; Sato et al., 2017), MS patient-derived iPSCs have been differentiated into neurons (Song et al., 2012), astrocytes (Song et al., 2012), oligodendrocyte precursors (Nicaise et al., 2017), and oligodendrocytes (Song et al., 2012; Douvaras et al., 2014). The only differences so far identified are an inability of MS iPSCNs to fire spontaneous action potentials (Song et al., 2012), and reduced neuroprotection by MS iPSC oligodendrocyte precursors (Nicaise et al., 2017). Microglia, which have been sparingly used to model neurological disease, have also been used to model Rhett syndrome (Muffat et al., 2016). The use of iPSCs to model neuropsychiatric disorders is expanding rapidly, as reviewed by Ho et al. (2015).

Reprogrammed patient-derived cells have been used to model sporadic and familial neurodegenerative, neurodevelopmental, and neuropsychiatric disease. Much early work using iPSCs to study neurological disease focused on validating previously-observed phenotypes from animal models and postmortem autopsy tissues. Now that the ability of iPSC-derived CNS models to recreate disease pathology has been established, more recent studies are beginning to use iPSCs to tease out disease mechanisms and screen for pre-clinical therapeutics. These efforts are supported by continuing improvements in cell type-specific differentiation methods, the expansion of co-culture and 3D culture models, and the generation of genetically corrected isogenic patient lines to account for the diversity of patients' intrinsic genetic backgrounds.

Despite the advantages and advancements of patient-derived iPSCs, concerns about differentiation time, culture specificity and purity, teratoma formation in transplantation, and, most importantly, the difficulty for rejuvenated cells to model aging diseases has led to the development of a complimentary methodology: lineage-specific direct conversion of patient-derived somatic cells.

## Direct conversion overview

One distinct advantage of direct lineage conversion, also known as transdifferentiation, is that there is no reprogramming step. The epigenetic identity of the starting cells remains after conversion, as opposed to the hypermethylation observed when somatic cells are rejuvenated into iPSCs (Cohen-Hadad et al., 2016). This retains “cellular memories.” Most conversion protocols feature mesoderm to ectoderm conversions—meaning fibroblasts to neurons. Due to the infancy of this field, the definition of an induced neuron (iN) has many meanings (Yang et al., 2011). Direct conversion can occur from fibroblasts (Vierbuchen et al., 2010), blood (Lee et al., 2015), urine cells (Zhang S. Z. et al., 2016), hepatocytes (Marro et al., 2011), and adipocyte progenitors (Yang Y. et al., 2013), which provides a wide variety of non-invasive starting materials for modeling patient disease.

The first report of direct lineage reprogramming by Vierbuchen et al. (2010) used combinatorial expression of three neural lineage-specific transcription factors—Brn2, Ascl1, and Myt1L, known as the BAM factors—to induce neurons from mouse fibroblasts (Vierbuchen et al., 2010). The induced neurons (hereafter called iNeurons or iNs) that result from this method express pan-neuronal markers including Tuj1, NeuN, MAP2, and synapsin. These iNs also fire induced and spontaneous action potentials and form functional synapses. However, the BAM factors alone cannot convert human fetal or postnatal fibroblasts into functional neurons. The addition of the basic helix-loop-helix transcription factor NeuroD1 to the BAM factor conversion protocol was found to be necessary for iNs to be produced from human somatic cells (Pang et al., 2011).

Here, we will first describe the general methods used to directly convert somatic cells. We then specify conversion methods applied to the generation of neurons and glial cells, followed by a summary on how these directly converted cells have been studied in varying neurodegenerative diseases thus far.

## Direct conversion methods

There are three primary methods of directly converting human somatic cells into CNS cells. Transcription factor overexpression, microRNA overexpression, and small molecule treatments can, individually or in concert, generate a variety of cell types from the aforementioned starting materials.

### Transcription factor-mediated direct conversion

Since Pang et al. first successfully created human iNs, a number of other groups have obtained similar results using some or all of the BAM transcription factors (Ambasudhan et al., 2011; Caiazzo et al., 2011; Pfisterer et al., 2011; Son et al., 2011). Furthermore, Marro et al. used the BAM factors to convert hepatocytes into functional neurons, providing the first proof that an endodermal cell can be converted into an ectodermal cell (Marro et al., 2011). Renal epithelial cells from urine can be directly converted into neurons via lentivirus-mediated expression of BAM factors plus NeuroD1 and c-Myc (Zhang S. Z. et al., 2016). Mitchell et al. (2014) showed the Oct4 expression alone was enough to generate NPCs from somatic cells. These are only a few examples of the variety of transcription factors used to generate neurons (Mitchell et al., 2014).

### Microrna-mediated direct conversion

Several groups have also achieved direct conversion via microRNAs, although concomitant expression of neural lineage-specific transcription factors is required to fully mature these iNs. Coexpression of miR-9/9^*^ and miR-124 is sufficient to directly convert human fibroblasts into neurons (Yoo et al., 2011). While initial conversion efficiency using these microRNAs was very low, efficiency increased with the addition of the transcription factors NeuroD2, Ascl1, and Myt1. Furthermore, striatal medium spiny neurons or motor neurons can also be generated by co-expression of miR-9/9^*^ and miR-124 in addition to the transcription factors BCL11B (CTIP12), DLX1, DLX2, and MYT1L or ISL1 and LHX3, respectively (Victor et al., 2014; Richner et al., 2015; Abernathy et al., 2017). Huh et al. also showed that direct conversion using this protocol preserves age-related epigenetic and cellular signatures from the donor, similar to established transcription factor-only methods (Huh et al., 2016). Primary dermal human fibroblasts can also be converted into functional neurons by expression of miR-124 in addition to transcription factors Brn2 and Myt1 (Ambasudhan et al., 2011). An additional method reported by Zhou et al. (2014) showed that depletion of p53 by zinc finger nuclease (ZFN) technology is sufficient to convert fibroblasts into neurons, oligodendrocytes, or astrocytes, depending on the selection media used (Zhou et al., 2014). It has not yet been established whether p53 depletion generates neurons which recapitulate disease pathology.

Similar to microRNA-mediated approaches, expression of short interfering RNA (siRNA) inhibiting the polypyrimidine-tract binding (PTB) protein has been shown to be sufficient for transdifferentiation (Xue et al., 2013). The suppression of PTB allows for expression of various neuronal genes, including the neuron specific microRNA-124. In non-neuronal cells, the PTB protein inhibits microRNAs which act on various components of the RE1-silencing transcription factor complex. Relief of this PTB-protein inhibition leads to activation of an array of neuronal lineage-specific genes, thereby promoting neuronal induction.

Lau et al. report using a self-regulating viral vector which expresses BAM factors and has target sequences for microRNA-124. Once the cells have been converted into neurons, they will express miR-124, and then downregulate the transgenes (Lau et al., 2014). The microRNA-mediated downregulation of the neural conversion genes allow for more complete functional maturation in culture.

MicroRNAs, with their ability to both promote expression of lineage specific genes and suppress expression after conversion, offer a path to fine-tune conversion to best replicate endogenous expression of cell-type specific genes and more accurately recreate their effect on disease biology in the patient. This benefit must be weighed against the fact that most microRNAs have a variety of target genes, and overexpression may cause unintended dysregulation of disease-relevant genes.

### Small molecule-mediated direct conversion

In addition to transcription factors, small molecules can convert human fibroblasts into functional neurons without the addition of exogenous genetic factors. The resulting neurons are called chemically induced neurons (CiNs). Small molecules have been shown to increase efficiency of conversions when combined with transcription factors (Ladewig et al., 2012; Liu et al., 2013; Lee et al., 2015; Mertens et al., 2015; Shi et al., 2016). SMAD, GSK3β, and ALK inhibitors, alongside cyclic AMP signaling enhancers, are common between CiN and iPSC differentiation. In an early study, (Ladewig et al., 2012) combined Ascl1 and Ngn2 with the small molecules SB-431542, noggin, and CHIR99021 (a GSK3β inhibitor). Reports of purely small molecule conversions include a combination of forskolin, isoxazole 9 (an inducer of neural stem cell differentiation), CHIR99021 and I-BET151 (a BET bromodomain inhibitor), which can convert fibroblasts into functional neurons that express pan-neuronal markers (Li X. et al., 2015), and a small molecule cocktail called VCR, consisting of Valproic acid, CHIR99021 and Repsox, which is capable of inducing NPCs (Cheng et al., 2014). Hu et al. expanded VCR and generated human CiNs using the cocktail named VCRFSGY, which consisted of Valproic acid, CHIR99021, Repsox, Forskolin, SP600125 (a JNK inhibitor) GO6983 (a PKC inhibitor) and Y-27632 (a ROCK inhibitor) (Hu et al., 2015). These hCiNs showed some spontaneous electrical activity and a train of action potentials when stimulated.

## Direct conversion cell types

Direct conversion has generated nearly as wide a variety of CNS cell types as have stem cell differentiation approaches. Proneural transcription factors without subtype specification largely result in glutamatergic and GABA-ergic iNs (Vierbuchen et al., 2010; Wang et al., 2014; Mertens et al., 2015). Success of specific cell type generation by direct conversion techniques are largely based on expression of cell type-specific markers, but the question remains if the final cell type generated mimics bona fide neuron subtypes. As in iPSC models, cell type confidence can be increased by considering subtype-specific morphology, gene expression, and electrophysiology in concert with marker proteins and in comparison to individual cell types of adult human tissue.

### Direct brain-native neuronal subtype conversion

Induced neural progenitor cells (iNPCs) can be generated via expression of pluripotency factors in possible combination with small molecules (Kim et al., 2011a; Mitchell et al., 2014; Lee et al., 2015; Hou et al., 2017). iNPCs are not post-mitotic, and can differentiate into various neuronal subtypes, oligodendrocytes, and astrocytes *in vivo* and *in vitro*. Pro-neural and serotonergic neuron-specific transcription factors, in addition to small molecules, can generate induced neurons that have characteristics of native serotonergic neurons such as release of serotonin, response to SSRIs, increased expression of specific serotonergic genes, and firing of spontaneous action potentials (Vadodaria et al., 2016; Xu et al., 2016). Dopaminergic neurons are of particular interest due to their clinical relevance in Parkinson's disease. It has been shown that induced DA neurons (iDAs) can be generated from human fibroblasts via a combination of transcription factors (Caiazzo et al., 2011; Kim et al., 2011b; Pfisterer et al., 2011; Liu et al., 2012; Torper et al., 2013; Dell'Anno et al., 2014). The resulting iDAs displayed characteristic DA uptake and release, expressed DA neuron-specific markers, and were electrically active.

### Direct motor neuron conversion

Transdifferentiation can also model nervous system disease outside of the brain. The small molecules Forskolin and Dorsomorphin, in addition to the transcription factors Neurogenin2 and SOX1, can generate human induced cholinergic neurons with mature electrophysiological properties, motor neuron-like features, and morphology (Liu et al., 2013). Human induced motor neurons (hiMNs), which form functional NMJs when cultured with primary mouse skeletal myotubes, can be generated via expression of Ngn2, SOX11, ISL1, and LHX3, in addition to the small molecules forskolin and dorsomorphin (Liu et al., 2016). Son et al. utilized a transcription factor-only (BAM factors plus motor neuron-specific transcription factors Lhx3, Hb9, and Isl1, and NeuroD1) approach to generate functional human spinal motor neurons (Son et al., 2011).

### Direct sensory neuron conversion

Induced sensory neurons can be generated by the transient co-expression of Brn3a with either Ngn1 or Ngn2 and exhibit properties of endogenous sensory neurons such as gene expression, Trk receptor expression, response to ligands, and morphology (Blanchard et al., 2015). Nociceptive sensory neurons with nociceptor-specific functional receptors can be converted from fibroblasts using BAM factors in addition to Isl2, Ngn1, and Klf7 (Wainger et al., 2015) or via OCT4 and a small molecule cocktail which inhibits SMAD and GSK-3β signaling (Lee et al., 2015).

### Direct glial conversion

Direct conversion can also produce glia. The transcription factors NFIA, NFIB, and SOX9 can convert native brain embryonic and postnatal mouse fibroblasts into astrocytes (iAstrocytes) (Caiazzo et al., 2015). Induced oligodendrocyte precursor cells (iOPCs) can be generated from mouse and rat fibroblasts by direct lineage conversion using transcription factors such as SOX10, Olig2, and Zfp536, Nkx6.2, Egr2 (Najm et al., 2013; Yang N. et al., 2013; Mazzara et al., 2017). These iOPCs can differentiate *in vivo* and myelinate sections of axons that are not myelinated. To date, there have been no reports of direct conversion of somatic cells into microglia. Further development of glial cell protocols will be required to simulate non-cell autonomous neurological disease.

## Direct conversion models of disease

The diversity of conversion methods and sub-type specific fates gives researchers considerable flexibility for modeling disease. Disease states have been repeatedly shown not to impede conversion into neurons. The use of iNs in studying disease is still new and somewhat limited (Figure 2).

### Amyotrophic lateral sclerosis

Lim et al. generated iNeurons via the inhibition of PTB method to investigate three mutations in the nuclear localization signal region of the gene Fused in Sarcoma (FUS), a causative ALS gene (Lim et al., 2016). The ALS iNeurons showed increased cytoplasmic FUS localization when compared to controls. Another report studying mechanisms of mutant FUS generated hiMNs converted from the fibroblasts of three ALS patients with known FUS mutations. FUS was mislocalized into the cytoplasm in all cases; however, only one of the three ALS patients used in this study had a FUS mutation known to increase cytoplasmic retention of FUS (Liu et al., 2016), emphasizing that the use of patient-derived DC models aides in the discovery of novel disease mechanisms. These FUS ALS hiMNs also showed dramatically reduced electrical activity, failed to form functional NMJs when co-cultured with primary mouse skeletal muscles, had shrunken somas, and were more susceptible to cell death when compared to controls. Control iMNs co-cultured with glia from SOD1^G93A^ mice undergo more cell death than those co-cultured with control glia, replicating previously reported non-cell autonomous effects (Son et al., 2011). Similarly, induced NPCs from one patient with a SOD1^A4V^ mutation and three patients with the C9orf72 repeat expansion were differentiated into iAstrocytes and were found to be toxic to co-cultured primary mouse neurons (Meyer et al., 2014). iNs converted from C9orf72 ALS patients by PTB inhibition showed RNA foci, poly(GP) inclusions and poly(PR) inclusions (Su et al., 2014). These reports suggest that DC models can replicate pathological hallmarks of multiple ALS subtypes in ways similar to iPSC-derived models.

### Alzheimer's disease

CiNs from four Alzheimer's disease patients with mutations in APP (V717I) or PSEN1 (I167del, A434T, or S169del) showed higher extracellular levels of Aβ40 and Aβ42 when compared to controls. iNs from AD patients also showed an increase in total tau and phosphorylated tau (Hu et al., 2015). Similar results regarding extracellular levels of Aβ40 and Aβ42 have been reported in induced NPCs differentiated into neurons from three AD patients, one with APOE4/E4 mutation and two with mutations in the PSEN1 gene (Hou et al., 2017).

### Huntington's disease

Liu et al. (2014) investigated the use of induced neuron technology to directly convert human fibroblasts from patients with Huntington's disease. Human fibroblasts from HD patients were converted by inhibition of PTB (Liu et al., 2014). The HD iNs exhibited nuclear mutant-Htt inclusions, abnormal neuritic branching, and lower cell survival. In another study, iNPCs were generated from HD patient fibroblasts and then further differentiated into neurons which exhibited higher vulnerability to DNA damage than controls (Hou et al., 2017).

### Spinal muscular atrophy

Spinal muscular atrophy (SMA) patient fibroblasts converted into hiMNs saw reduction in neurite growth rate and, after 60 days *in vitro*, degeneration of the neurites (Zhang et al., 2017). Induced neurons from patients with mutations in the PTEN-induced putative kinase 1 (PINK1) pathway, a genetic risk factor for Parkinson's disease, show reduction of downstream targets of this pathway which are involved in regulation of mitochondrial quality control (Puschmann et al., 2017).

Direct conversion cells have demonstrated the ability to mimic disease pathology and physiology in a number of neurological disorders (Figure 2). What has not been established yet is whether or not the retention of epigenetic signatures allows DC models to recreate disease pathology more accurately than rejuvenated iPSC models and whether cells generated from the same patient using both differentiation methods would display different disease phenotypes.

## Concluding remarks

**Figure 3:**
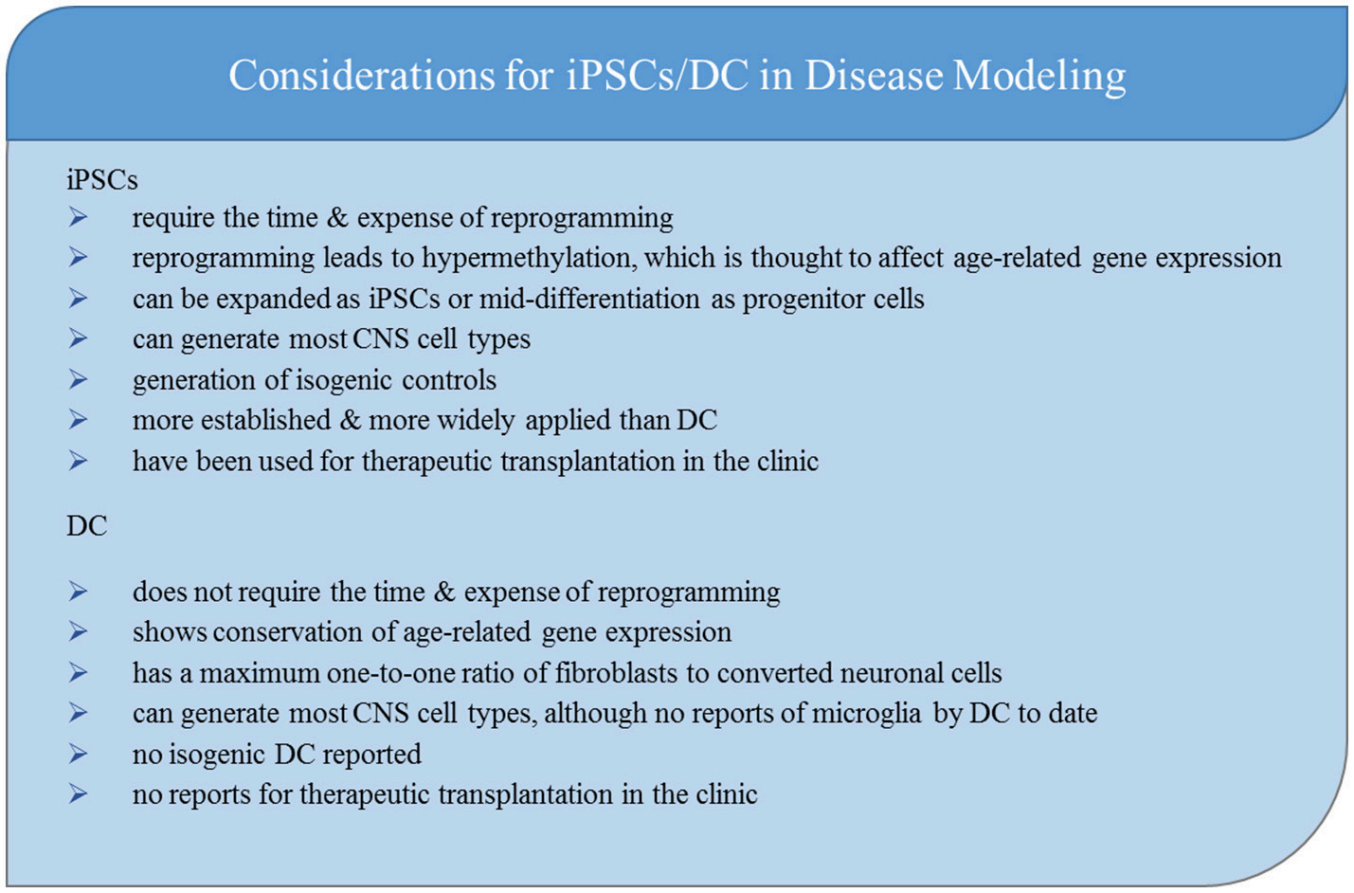
Key considerations for choosing iPSCs or DC to model neurological disease.

Patient-derived CNS models have expanded rapidly in the past decade. A variety of adult somatic cells can be reprogrammed into iPSC's and differentiated or directly converted into the cells necessary to model disease, test novel drug candidates, and be used for cell transplantations. As researchers today design new studies of familial and sporadic neurological conditions, they must carefully consider which model and method most efficiently answers their scientific question (Figure 3).

Patient-derived CNS models are time intensive. While direct conversion decreases the time to generate neurons from somatic sample collection due to the lack of reprogramming, the time it takes to differentiate and mature functional neurons from fibroblasts or iPSCs is similar. iPSC-based models offer more efficient production of cells than direct conversion because cells are infinitely expandable in both the iPSC stage and the NPC stage. While the yield of the desired cell type (e.g., neuron vs. astrocyte) is high, sub type (e.g., ChAT+ vs. CTIP2+) yield can vary widely between different small molecule and growth factor-based differentiation methods. Virally-expressed transcription factors may increase specific cell subtype yield in iPSC differentiations. While fibroblasts used for direct conversion are infinitely expandable, the lack of an intermediate progenitor stage creates a maximum one-to-one ratio of starting fibroblasts to desired cell type. In addition, there are reports of desired neuronal subtype yields of fewer than 10% (Vierbuchen et al., 2010; Pfisterer et al., 2011). Given the time and expense of generating the cells, this low yield must be weighed against the scientific benefits of aging retention in the model.

One application for patient-derived cells which has not been addressed at length in this review, but has been recently reviewed by Marsh and Blurton-Jones (2017), Czarzasta et al. (2017), Wang et al. (2017), and Smith et al. (2017), is the ability to implant iPSC-derived or directly converted NPCs and differentiate them *in vivo* to perform neurotrophic roles or replace cells lost through degeneration.

While comparative analyses of epigenetics and gene expression (particularly age-related genes) in iNeurons and iPSNs have demonstrated the loss of aging signatures in reprogramming (Mertens et al., 2015; Huh et al., 2016), the effects of these expression changes on the ability to model disease is poorly understood. Whether or not certain disease hallmarks, including proteinopathies, morphological and functional changes, and differences in survival in response to stressors will manifest differently when epigenetic signatures are preserved remains to be seen. A recent comparison of methylation of the C9orf72 transcript in repeat expanded eSCs and iPSCs suggests that toxic RNA transcription, and thus presumably RAN DPR translation, is attenuated in iPSCs and may suppress the role these mechanisms play in disease (Cohen-Hadad et al., 2016). This supports the argument that reprogramming will have an effect on disease pathology.

The clonal nature of iPSCs allows for the generation of CRISPR-Cas9 correction of disease-causing mutations, which can help establish whether certain pathologies observed in the model are attributable to the mutation and provides an isogenic control. This technique is not currently available in direct conversion; however, there may be some advantage to generating model neurons from a genetically and epigenetically heterogeneous population of somatic cells rather than from copies of a single iPSC clone, especially given the heterogeneity of disease pathology between cells in patient tissue.

Both direct conversion and iPSC differentiation have produced glia and several neuronal subtypes. Specific differentiation methods, including transcription factor choice, small molecule and growth factor choice, and plating methods have been and will continue to be adjusted to create a higher yield of desired cell types in less time. While, due to its earlier discovery, iPSC differentiation technology is currently better explored and developed, it is likely that iNeurons and other direct conversion cell types will be equally fine-tuned in the near future. With either method, care should be taken in regards to the true identity of generated cell types, given the extensive cellular conversion these cells undergo. Genetic and proteomic analyses of differentiated or converted cell types, along with comparisons to postmortem cell-type specific analyses, can address some of these concerns.

The extensive reports of disease pathologies and pathogenesis summarized in this review suggest that, despite the limitations of *in vitro* human cell culture models, they are capable of recreating disease-specific pathologies. In addition, new disease pathways have been discovered using patient-derived cell culture models which were then validated in postmortem patient tissue, such as the presence of nucleocytoplasmic transport deficits and nuclear pore dysfunction in ALS, FTD, HD and, likely, in other neurodegenerative diseases. It is therefore likely that other novel disease mechanisms will be revealed using these human cell culture models.

Patient-derived cells offer hope for an endogenous, human model of neurological disease, especially for sporadic cases which cannot be modeled any other way, and provide a platform for rapid therapeutic discoveries that are both personalized and clinically translatable.

## Author contributions

LG, AS, AN, and RS: conceptualized and wrote the manuscript.

### Conflict of interest statement

The authors declare that the research was conducted in the absence of any commercial or financial relationships that could be construed as a potential conflict of interest.
